# A Clinician’s Guide to Cancer Therapy-Related Cardiovascular Toxicity

**DOI:** 10.31083/RCM54342

**Published:** 2026-10-16

**Authors:** Mukul Bhattarai

**Affiliations:** ^1^Cardio-Oncology and Amyloid Program, Division of Cardiology, Department of Internal Medicine, Southern Illinois University School of Medicine, Springfield, IL 62702, USA

**Keywords:** cardio-oncology, cardiotoxicity, anthracyclines, immune checkpoint inhibitors, tyrosine kinase inhibitors, radiation-induced heart disease, cardiac amyloidosis, cardiovascular imaging, cancer survivorship, cardiomyopathy

## Abstract

Advances in cancer therapy have substantially improved survival, resulting in a rapidly growing population of cancer survivors. Consequently, cardiovascular disease has emerged as a major determinant of long-term morbidity and mortality, establishing cancer therapy-related cardiovascular toxicity as a central challenge in contemporary cardio-oncology. Modern oncologic therapies produce a broad spectrum of cardiovascular complications, including heart failure, arrhythmias, myocarditis, vascular toxicity, thromboembolic disease, valvular abnormalities, pulmonary hypertension, and autonomic dysfunction. These toxicities arise through diverse mechanisms, including direct myocardial injury, endothelial dysfunction, immune-mediated inflammation, cytokine excess, metabolic derangements, and neurohormonal alterations. This review provides a clinician-oriented, mechanism-based overview of cancer therapy-related cardiovascular toxicity in the modern era. Major therapeutic classes are discussed, including anthracyclines, human epidermal growth factor receptor 2 (HER2)-targeted therapies, tyrosine kinase inhibitors, immune checkpoint inhibitors (ICIs), chimeric antigen receptor T cell therapies, radiation therapy, endocrine therapies, and emerging targeted agents. The review also highlights evolving cardiovascular toxicity phenotypes, radiation-induced heart disease, and the growing relevance of cardiac amyloidosis within the cardio-oncology spectrum. Moreover, contemporary strategies for cardiovascular risk assessment, imaging and biomarker surveillance, cardioprotection, and therapy-specific management are reviewed, with an emphasis on practical clinical application. Finally, current knowledge gaps, survivorship considerations, and future directions are discussed, including artificial intelligence-assisted risk stratification. Overall, by integrating mechanistic understanding with practical management frameworks, this review aims to provide clinicians with a comprehensive resource for the prevention, detection, and treatment of cardiovascular complications associated with modern cancer therapies.

## 1. Introduction: The Modern Cardio-Oncology Landscape

Advances in cancer detection and therapy have led to substantial improvements in survival across a wide range of malignancies, resulting in a rapidly expanding population of cancer survivors worldwide. As survival has improved, cardiovascular disease has emerged as a leading cause of long-term morbidity and mortality among cancer survivors, in some cohorts surpassing the risk of recurrent malignancy [1,2,3]. This epidemiologic shift has fundamentally altered the landscape of cancer survivorship and established cancer therapy–related cardiovascular toxicity as a major determinant of long-term outcomes.

Cancer therapy–related cardiovascular toxicity encompasses a broad spectrum of clinical phenotypes, including left ventricular systolic dysfunction, heart failure with preserved ejection fraction, arrhythmias, ischemic heart disease, thromboembolic events, valvular disease, pericardial pathology, pulmonary hypertension, and autonomic dysfunction [4]. These manifestations may occur acutely during active treatment, subacutely in the months following therapy, or years to decades after exposure, particularly among patients treated at a younger age. This clinical heterogeneity reflects distinct underlying mechanisms, including direct myocardial injury, endothelial dysfunction, immune-mediated inflammation, metabolic derangements, and neurohormonal dysregulation (Fig. 1).

**Fig. 1.**
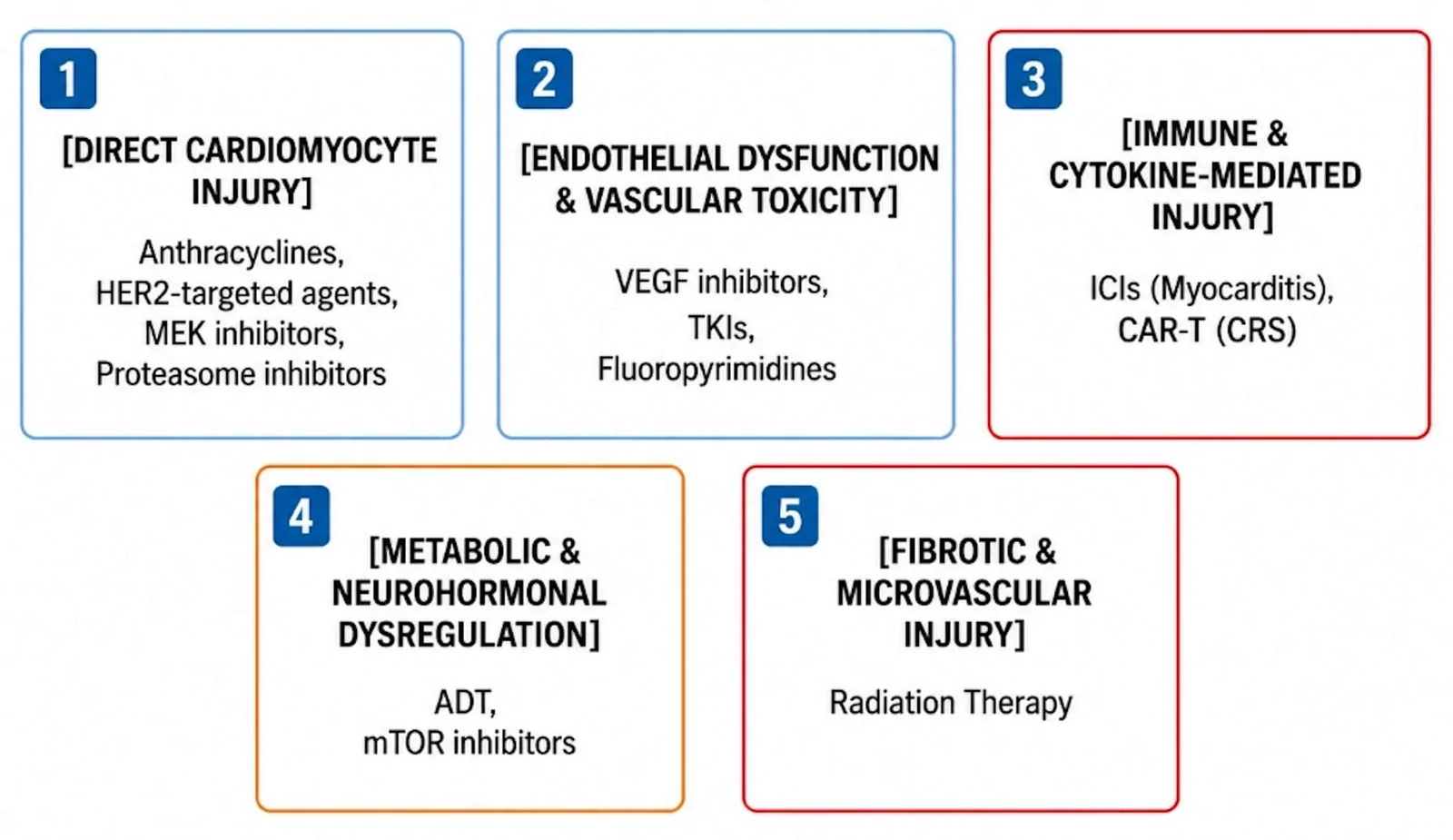
**Mechanism-based classification and representative antineoplastic drug classes of cancer therapy-related cardiovascular toxicities**. HER2, human epidermal growth factor receptor 2; VEGF, vascular endothelial growth factor; TKIs, tyrosine kinase inhibitors; ICIs, immune checkpoint inhibitors; CAR-T, chimeric antigen receptor T-cell; CRS, cytokine release syndrome; ADT, androgen deprivation therapy; MEK, mitogen-activated protein kinase.

The contemporary oncology landscape is increasingly dominated by targeted therapies, immune-based treatments, and cellular therapies. While these advances have transformed cancer outcomes, they have introduced cardiovascular toxicity profiles that differ substantially from those associated with traditional cytotoxic chemotherapy [5]. Tyrosine kinase inhibitors (TKIs) are frequently associated with hypertension, vascular toxicity, and heart failure; immune checkpoint inhibitors (ICIs) may precipitate fulminant myocarditis and conduction system disease; and chimeric antigen receptor T-cell therapies are commonly complicated by cytokine-mediated cardiovascular dysfunction [6,7]. These emerging phenotypes challenge traditional cardiology paradigms and require a mechanism-based, therapy-specific approach to cardiovascular risk assessment.

Despite growing recognition of these toxicities, standardized approaches to surveillance, prevention, and management remain variably implemented across clinical practice. Contemporary guidelines emphasize integration of cardiac imaging, circulating biomarkers, and longitudinal clinical assessment to enable early detection and timely intervention [8]. The European Society of Cardiology – International Cardio-Oncology Society (ESC–ICOS) monitoring and management framework for cancer therapy–related cardiac dysfunction (CTRCD) is illustrated in Fig. 2 (Ref. [8]). However, important evidence gaps remain regarding optimal monitoring intervals, thresholds for cardioprotective therapy, and long-term cardiovascular risk stratification in patients receiving modern oncologic agents.

**Fig. 2.**
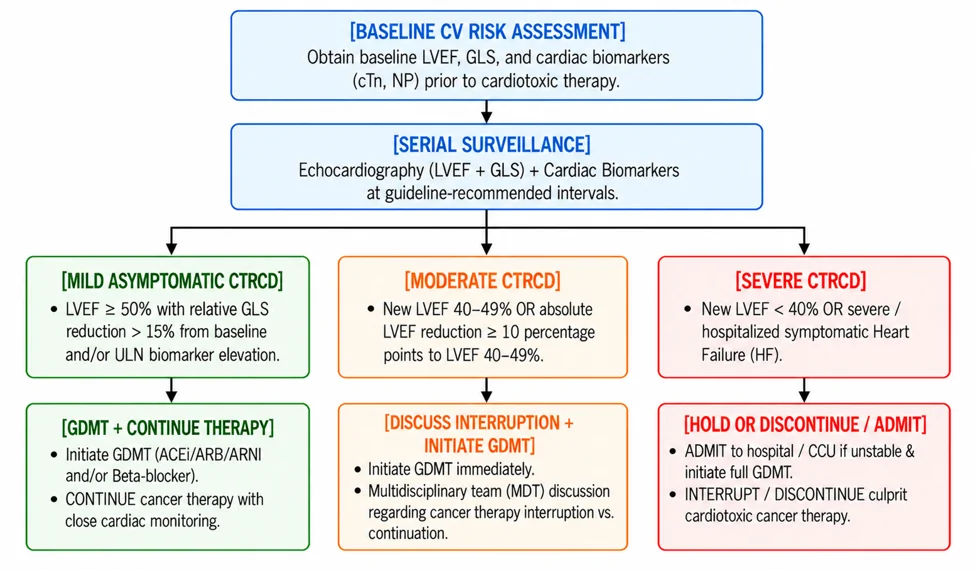
**ESC/ICOS-aligned monitoring & management algorithm for CTRCD**. Fig. 2 was adapted from 2022 ESC/ICOS Guidelines [8]. ESC, European Society of Cardiology; ICOS, International Cardio-Oncology Society; CTRCD, cancer therapy–related cardiac dysfunction; CV, cardiovascular; LVEF, left ventricular ejection fraction; GLS, global longitudinal strain; ULN, upper limit of normal; HF, heart failure; GDMT, guideline-directed medical therapy; ACEi, angiotensin-converting enzyme inhibitor; ARB, angiotensin receptor blocker; ARNI, angiotensin receptor–neprilysin inhibitor; MDT, multidisciplinary team; CCU, cardiac care unit.

Accordingly, there is a critical need for clinician-oriented frameworks that integrate mechanistic understanding with practical guidance. In this State-of-the-Art Review, we present a comprehensive, mechanism-based overview of cancer therapy–related cardiovascular toxicities in the modern era, incorporating drug-specific phenotypes, emerging cardiovascular syndromes, and practical strategies for monitoring, prevention, and management.

## 2. Pathophysiologic Framework of Cardiotoxicity

Cancer therapy–related cardiovascular toxicity arises from distinct but often overlapping pathophysiologic mechanisms that reflect both direct myocardial injury and systemic effects of oncologic therapies. Unlike traditional cardiovascular disease, which evolves over decades, treatment-related cardiotoxicity may develop acutely, subacutely, or years after exposure, depending on therapeutic class, cumulative dose, and host susceptibility. A mechanism-based framework is therefore essential to guide risk assessment, surveillance, and management.

### 2.1 Direct Myocardial Injury and Reduced Reserve

Direct cardiomyocyte injury represents a foundational mechanism of cardiotoxicity, most classically associated with anthracycline exposure. Anthracyclines induce myocardial damage through inhibition of topoisomerase-2β, leading to DNA double-strand breaks, mitochondrial dysfunction, and reactive oxygen species generation, culminating in irreversible myocyte loss and reduced myocardial reserve [9,10].

Importantly, apparent recovery of left ventricular ejection fraction does not reflect true myocardial recovery, as structural and energetic deficits persist, predisposing patients to future heart failure and increased vulnerability to additional cardiotoxic therapies [11].

Other agents, including high-dose cyclophosphamide and proteasome inhibitors, may produce acute myocardial injury characterized by rapid onset systolic dysfunction, myocardial edema, and capillary leak within days of exposure [12].

### 2.2 Endothelial Dysfunction and Vascular Injury

Endothelial dysfunction is a dominant mechanism underlying cardiovascular toxicity associated with targeted therapies, particularly vascular endothelial growth factor (VEGF) pathway inhibitors. Inhibition of VEGF signaling reduces nitric oxide bioavailability, promotes vasoconstriction, and induces microvascular rarefaction, resulting in hypertension, arterial thrombosis, and heart failure [13,14].

These vascular effects often occur early during therapy and may precede overt myocardial dysfunction. Chronic endothelial injury also accelerates atherosclerosis and contributes to long-term cardiovascular risk in cancer survivors.

### 2.3 Immune-Mediated Cardiac Inflammation

ICIs introduce a distinct cardiotoxicity paradigm driven by immune-mediated inflammation. ICI-associated myocarditis is characterized by T-cell infiltration of the myocardium and conduction system, leading to arrhythmias, conduction abnormalities, and, in severe cases, fulminant heart failure [6,15].

Notably, myocardial inflammation may occur in the absence of significant left ventricular dysfunction, highlighting the importance of biomarker and electrocardiographic monitoring. Additional immune-mediated manifestations include pericarditis, vasculitis, and systemic inflammatory syndromes.

### 2.4 Cytokine-Mediated Cardiovascular Dysfunction

Cytokine release syndrome (CRS), most commonly associated with chimeric antigen receptor T-cell (CAR-T) therapy, represents a unique mechanism of cardiovascular toxicity. Excessive cytokine release, particularly interleukin-6, results in systemic vasodilation, capillary leak, myocardial stunning, and hemodynamic instability [16].

Cardiac manifestations include tachyarrhythmias, transient left ventricular dysfunction, and troponin elevation. Unlike anthracycline-induced injury, cardiac dysfunction in this setting is typically reversible with appropriate cytokine-directed therapy.

### 2.5 Metabolic, Neurohormonal, and Autonomic Effects

Several cancer therapies induce metabolic and neurohormonal alterations that indirectly increase cardiovascular risk. Androgen deprivation therapy and endocrine treatments promote insulin resistance, dyslipidemia, and adverse body composition changes, accelerating atherosclerosis and heart failure risk [17].

Radiation therapy and select systemic treatments may also disrupt autonomic regulation, leading to impaired heart rate recovery, reduced exercise capacity, and increased arrhythmic susceptibility. These changes contribute to long-term cardiovascular morbidity, particularly in cancer survivors exposed to mediastinal radiation.

### 2.6 Overlapping and Multimodality Mechanisms

In clinical practice, cardiotoxic mechanisms frequently overlap, particularly in patients receiving multimodality therapy. Sequential exposure to anthracyclines, targeted therapies, and radiation may result in compounded myocardial injury, impaired compensatory signaling, and progressive loss of myocardial reserve.

Recognition of these interacting mechanisms is critical for individualized risk stratification and underscores the importance of a mechanism-based approach to cardio-oncology care.

## 3. Drug-Class–Specific Cardiovascular Toxicities

Cancer therapies produce cardiovascular toxicities that cluster predictably according to molecular target and downstream signaling pathways. Organizing toxicities by drug class and mechanism, rather than by cancer type, allows clinicians to anticipate adverse effects, tailor surveillance, and intervene early. The following section provides a mechanism-based synthesis aligned with the Master Cardio-Oncology Toxicity Table (Table 1) and visually integrated within a unified mechanistic framework (Fig. 1).

**Table 1.** **Mechanism-based master table of cancer therapy–related cardiovascular toxicities**.

Therapy class	Agent(s)	Primary molecular target	Key cardiovascular toxicities	Mechanism	Clinical pearls / monitoring
Anthracyclines	Doxorubicin, Epirubicin	Topoisomerase-2β	Irreversible LV systolic dysfunction, HF	Myocyte necrosis via ROS and DNA damage	Risk increases ≥250 mg/m^2^; do not rechallenge after LVEF recovery; dexrazoxane ≥300 mg/m^2^
HER2 inhibitors	Trastuzumab, Pertuzumab	HER2	Reversible cardiomyopathy	Impaired myocardial survival signaling	Hold if EF <50% with ≥10% drop; resume once EF ≥50%
VEGF TKIs	Sunitinib, Sorafenib, Pazopanib, Cabozantinib	VEGFR	Severe HTN, arterial thrombosis, HF	Endothelial dysfunction, reduce NO, capillary rarefaction	BP weekly ×6 weeks; aggressive HTN control mandatory
EGFR TKIs	Osimertinib	EGFR	QT prolongation, cardiomyopathy	Ion-channel interference, myocyte toxicity	Baseline ECG; hold if QTc ≥500 ms
BTK inhibitors	Ibrutinib, Zanubrutinib	BTK / TEC	HTN, AF, bleeding	Irreversible platelet dysfunction, atrial remodeling	Avoid non-DHP CCBs and amiodarone (CYP3A4 interaction)
MEK inhibitors	Trametinib, Cobimetinib, Binimetinib	MEK1/2	Reversible reduce LVEF	MAPK pathway inhibition	“-metinib = MEK = EF drop”; recovery with interruption
BCR–ABL TKIs	Ponatinib, Dasatinib, Nilotinib	BCR–ABL	Arterial thrombosis, PAH, PAD	Endothelial injury, accelerated atherosclerosis	Ponatinib = highest arterial risk; Dasatinib → PAH
CDK4/6 inhibitors	Ribociclib	CDK4/6 (hERG)	QT prolongation	hERG potassium-channel blockade	Hold if QTc ≥480 ms; ECG Day 0, 14, 28
Immune checkpoint inhibitors	Pembrolizumab, Nivolumab, Ipilimumab	PD-1 / CTLA-4	Myocarditis, arrhythmias, AV block	T-cell myocardial infiltration	Same-day IV steroids; troponin-driven diagnosis
CAR-T therapy	Axicabtagene, Tisagenlecleucel	CD19	CRS, acute HF, arrhythmias	IL-6–mediated myocardial stunning	Treat CRS with tocilizumab; EF usually recovers
Radiation therapy	Mediastinal RT	DNA / microvasculature	CAD, VHD, restrictive CMP	Fibrosis, microvascular endothelial injury	Begin screening 5 yrs post-RT; ostial / proximal LAD disease
Fluoropyrimidines	5-FU, Capecitabine	Thymidylate synthase	Coronary vasospasm, MI, arrhythmias	Endothelial NO depletion, vasoreactivity	Chest pain may occur with normal ECG; stop drug if suspected
APL therapy (retinoids / ATO)	ATRA, Arsenic trioxide	RAR-α / PML-RARα	Differentiation syndrome, QT prolongation	Cytokine release; hERG blockade (arsenic)	Treat DS with IV dexamethasone; hold arsenic if QTc ≥500 ms
Hormonal / ADT	Leuprolide, Goserelin, Degarelix	GnRH axis	Dyslipidemia, insulin resistance, increase cardiovascular risk	Hypogonadism-mediated metabolic derangement	Lipids worsen within 3 months; Degarelix may reduce MACE vs agonists
Androgen receptor inhibitors	Enzalutamide	Androgen receptor	HTN, ischemic events (rare)	Off-target vascular and metabolic effects	Monitor BP and falls risk
mTOR inhibitors	Everolimus, Sirolimus, Temsirolimus	mTOR	Dyslipidemia, edema, HTN, ILD	Metabolic dysregulation, endothelial effects	“-limus = lipids + lungs”; monitor TGs and pulmonary symptoms
Aromatase inhibitors	Anastrozole, Letrozole, Exemestane	Aromatase enzyme (CYP19A1) reduces peripheral estrogen synthesis	Accelerated atherosclerotic CVD, dyslipidemia, HTN, ischemic heart disease	Reducedperipheral estrogen synthesis No direct myocardial toxicity CV risk via atherosclerosis	Used in post menopause ER+ / PR+ breast cancer as adjuvant or long-term endocrine therapy. CV risk is primarily atherosclerotic. Monitor lipid profile, BP, metabolic risk factors, and symptoms of ischemia during long-term therapy.

Abbreviations: AF, atrial fibrillation; APL, acute promyelocytic leukemia; BTK, Bruton tyrosine kinase; CAD, coronary artery disease; CAR-T, chimeric antigen receptor T-cell; CDK, cyclin-dependent kinase; CMP, cardiomyopathy; CRS, cytokine release syndrome; EF, ejection fraction; EGFR, epidermal growth factor receptor; HER2, human epidermal growth factor receptor 2; HF, heart failure; hERG, human ether-à-go-go–related gene; HTN, hypertension; IL-6, interleukin-6; LV, left ventricular; MEK, mitogen-activated protein kinase; NO, nitric oxide; PAH, pulmonary arterial hypertension; PAD, peripheral artery disease; PD-1, programmed cell death protein-1; QTc, corrected QT interval; TEC, tyrosine kinase expressed in hepatocellular carcinoma; TKIs, tyrosine kinase inhibitors; VEGFR, vascular endothelial growth factor receptor; VHD, valvular heart disease; BCR–ABL, breakpoint cluster region–Abelson murine leukemia viral oncogene homolog; ATO, arsenic trioxide; ADT, androgen deprivation therapy; ATRA, all-trans retinoic acid; 5-FU, 5-fluorouracil; CTLA-4, cytotoxic T-lymphocyte–associated protein 4; CD19, cluster of differentiation 19; RAR-α, retinoic acid receptor alpha; PML, promyelocytic leukemia protein; GnRH, gonadotropin-releasing hormone; CYP19A1, cytochrome P450 family 19 subfamily A member 1; CVD, cardiovascular disease; ILD, interstitial lung disease; QT, QT interval; MI, myocardial infarction; AV, atrioventricular; ROS, reactive oxygen species; BP, blood pressure; ECG, electrocardiogram; DHP, dihydropyridine; CCBs, calcium channel blockers; CYP3A4, cytochrome P450 family 3 subfamily A member 4; LAD, left anterior descending coronary artery; DS, differentiation syndrome; IV, intravenous; MACE, major adverse cardiovascular events; TGs, triglycerides; ER, estrogen receptor; PR, progesterone receptor.

### 3.1 Tyrosine Kinase Inhibitors (TKIs)

#### 3.1.1 VEGF / Vascular Endothelial Growth Factor Receptor (VEGFR) Pathway Inhibitors

Vascular endothelial growth factor signaling plays a central role in endothelial homeostasis, nitric oxide production, and microvascular integrity. Inhibition of this pathway leads to vasoconstriction, capillary rarefaction, and increased systemic vascular resistance, manifesting clinically as hypertension, arterial thrombosis, and heart failure [13].

Hypertension is the most common and earliest cardiovascular toxicity associated with VEGF inhibitors and is often dose dependent. Failure to control blood pressure substantially increases the risk of downstream left ventricular dysfunction and ischemic events [14]. Agents such as sunitinib, pazopanib, cabozantinib, and sorafenib share this class effect, though the severity and associated thrombotic risk vary by compound.

Arterial thrombotic events—including myocardial infarction, stroke, and peripheral arterial disease—are particularly notable with pazopanib and ponatinib due to combined endothelial injury and prothrombotic signaling. Chronic exposure accelerates atherosclerosis and contributes to long-term cardiovascular morbidity in cancer survivors.

#### 3.1.2 Epidermal Growth Factor Receptor (EGFR) Tyrosine Kinase Inhibitors

Among epidermal growth factor receptor inhibitors, osimertinib occupies a unique position due to its dual association with QT prolongation and cardiomyopathy. Osimertinib inhibits human ether-à-go-go–related gene (hERG) potassium channels and disrupts myocardial signaling pathways, resulting in repolarization abnormalities and, in rare cases, reduced left ventricular ejection fraction [18].

Earlier-generation EGFR inhibitors such as erlotinib and gefitinib have minimal direct cardiotoxicity, underscoring the importance of distinguishing class effects from agent-specific toxicity.

#### 3.1.3 BTK Inhibitors

Bruton tyrosine kinase (BTK) inhibitors have transformed the management of B-cell malignancies but are strongly associated with atrial fibrillation and bleeding complications. Ibrutinib irreversibly inhibits BTK and tyrosine kinase expressed in hepatocellular carcinoma (TEC) kinases, impairing platelet aggregation and predisposing patients to hemorrhage [19].

Atrial fibrillation occurs in up to 15% of patients treated with ibrutinib and may present early or late during therapy. Hypertension is increasingly recognized as an additional contributor to long-term cardiovascular risk in this population [19]. Second-generation agents such as acalabrutinib and zanubrutinib appear to have lower arrhythmic risk but retain class effects.

#### 3.1.4 Anaplastic lymphoma kinase (ALK) and ROS proto-oncogene 1, receptor tyrosine kinase (ROS1) Inhibitors

ALK inhibitors such as crizotinib commonly cause sinus bradycardia through autonomic and direct nodal effects [20]. This bradycardia is typically asymptomatic but may necessitate dose reduction or temporary interruption in symptomatic patients. Lorlatinib, in contrast, is associated with metabolic derangements, including hyperlipidemia and hypertriglyceridemia, which indirectly increase cardiovascular risk.

#### 3.1.5 Mitogen-Activated Protein Kinase (MEK) Inhibitors (“-metinib” Agents)

MEK inhibitors—trametinib, cobimetinib, and binimetinib—are uniquely associated with reversible left ventricular systolic dysfunction. Inhibition of the MAPK pathway disrupts cardiomyocyte survival signaling without inducing permanent myocyte loss, distinguishing this toxicity from anthracycline cardiomyopathy [21].

Left ventricular dysfunction typically improves with drug interruption and guideline-directed medical therapy, allowing safe rechallenge in selected patients.

### 3.2 Monoclonal Antibodies and Targeted Biologics

#### 3.2.1 Human Epidermal Growth Factor Receptor 2 (HER2)-Targeted Therapies

Trastuzumab causes cardiomyopathy through inhibition of HER2-mediated cardiomyocyte survival pathways rather than direct cell death [22]. Unlike anthracyclines, trastuzumab-associated cardiomyopathy is typically reversible with treatment interruption and neurohormonal blockade.

Risk is highest when trastuzumab follows anthracycline exposure, reflecting loss of myocardial reserve. Current guidelines recommend echocardiographic surveillance every 3 months during therapy regardless of baseline risk.

#### 3.2.2 VEGF-Targeted Antibodies

Bevacizumab mirrors VEGF TKIs toxicity, producing hypertension, arterial thrombosis, and heart failure through endothelial dysfunction [23]. Wound-healing complications further complicate perioperative management.

### 3.3 Novel Cellular and Immune Therapies

#### 3.3.1 Immune Checkpoint Inhibitors (ICIs)

ICI-associated myocarditis represents the most lethal cardiotoxicity in modern oncology. T-cell infiltration of myocardium and conduction tissue leads to arrhythmias, heart block, and sudden death, often with preserved systolic function [6].

Troponin elevation and conduction abnormalities are often the earliest findings. Current ESC–ICOS guidelines recommend prompt initiation of high-dose intravenous corticosteroids when ICI-associated myocarditis is strongly suspected, without delaying treatment while awaiting confirmatory testing, as illustrated in Fig. 3 (Ref. [8]).

**Fig. 3.**
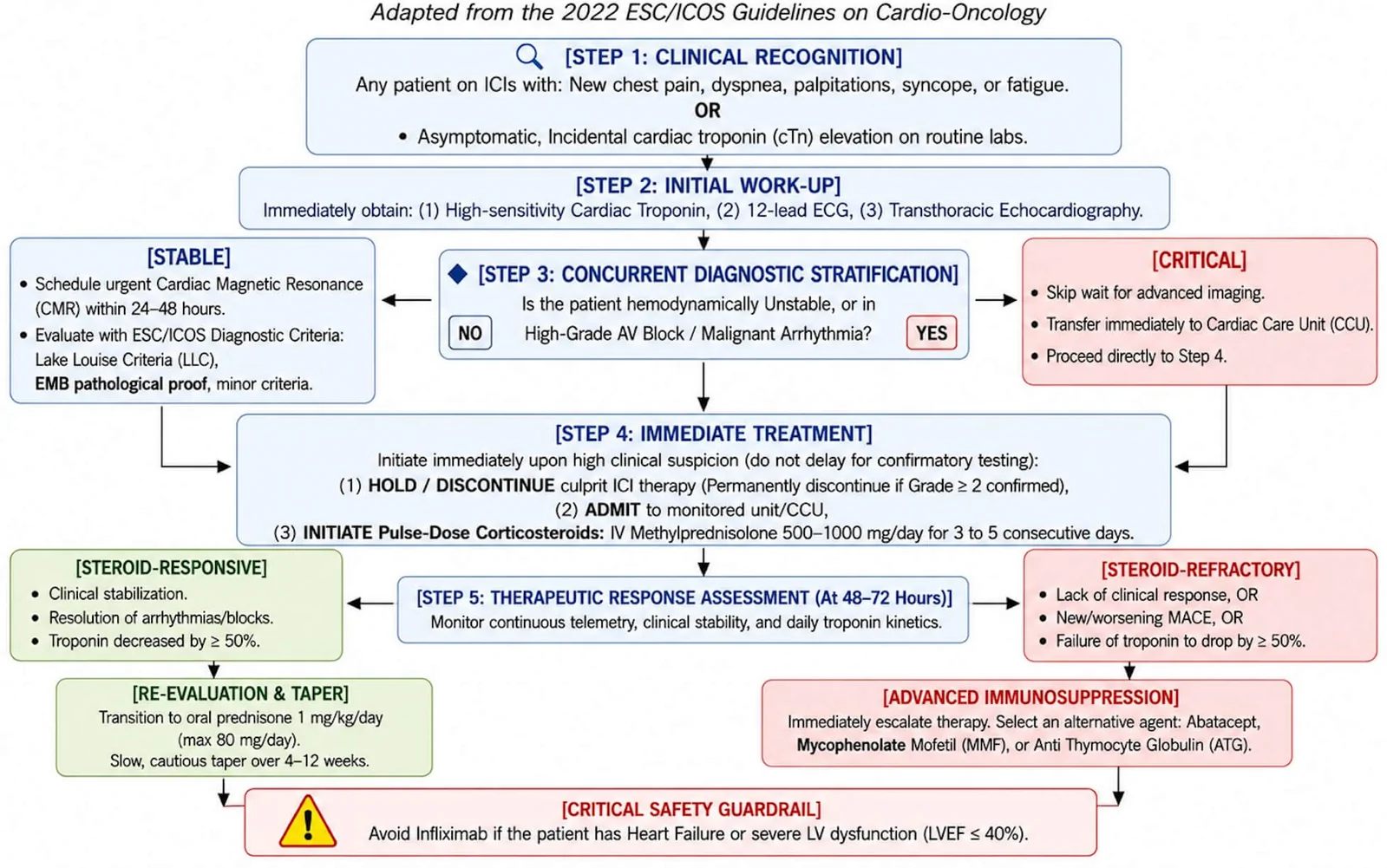
**Diagnostic & treatment approach for ICI-associated myocarditis**. Fig. 3 was adapted from 2022 ESC/ICOS Guidelines [8]. ICIs, immune checkpoint inhibitors; ECG, electrocardiogram; EMB, endomyocardial biopsy.

#### 3.3.2 Chimeric Antigen Receptor T-Cell (CAR-T)

Cardiovascular toxicity during CAR-T therapy is largely mediated by CRS. Interleukin-6–driven inflammation causes tachycardia, hypotension, myocardial stunning, and transient left ventricular (LV) dysfunction [7].

Prompt treatment with tocilizumab reverses hemodynamic compromise in most cases, distinguishing CRS-related dysfunction from immune-mediated myocarditis.

## 4. Emerging Cardiovascular Toxicity Phenotypes

The rapid evolution of oncologic therapeutics has introduced new cardiovascular toxicity phenotypes that extend beyond traditional cardiomyopathy and ischemia. Novel agents increasingly disrupt immune regulation, intracellular metabolism, autonomic signaling, and vascular integrity, creating cardiovascular syndromes that may be acute, delayed, or mechanistically distinct from classic chemotherapy-related injury. Recognition of these emerging patterns is essential to prevent catastrophic outcomes and to guide individualized surveillance strategies [24].

### 4.1 Beyond Classic ICI Myocarditis: Subclinical Neuro-Cardiac Injury

While ICIs associated myocarditis remains the most recognized immune-mediated cardiotoxicity, expanding evidence suggests a broader spectrum of immune-related cardiac injury. Subclinical myocardial inflammation, conduction disease without overt myocarditis, and overlap syndromes involving myositis and myasthenia gravis have been increasingly described [25].

Patients may present with modest troponin elevation, new conduction abnormalities, or ventricular arrhythmias in the absence of left ventricular systolic dysfunction. Cardiac magnetic resonance imaging may demonstrate myocardial edema without late gadolinium enhancement, reflecting early inflammatory injury rather than fibrosis.

Clinical implication: Any troponin elevation in patients receiving ICIs—particularly when accompanied by electrocardiogram (ECG) abnormalities—should prompt immediate evaluation and consideration of empiric corticosteroid therapy.

### 4.2 Delayed Cardiomyopathy Following Cellular Therapies

Delayed cardiomyopathy has emerged as a distinct entity following CAR-T therapy and immune-based regimens. Unlike acute CRS-mediated myocardial stunning, delayed dysfunction may occur weeks to months after therapy and is hypothesized to reflect immune-mediated myocardial injury or sustained inflammatory signaling [26].

This phenotype is characterized by gradual decline in left ventricular function, absence of fever or hypotension, and variable response to standard heart failure therapies. Long-term outcomes remain poorly defined, highlighting an important knowledge gap in survivorship care.

### 4.3 Metabolic Toxicity and Accelerated Atherosclerosis

Several modern targeted therapies produce profound metabolic derangements that indirectly amplify cardiovascular risk. ALK inhibitors such as lorlatinib and androgen receptor–targeted therapies such as enzalutamide and abiraterone induce hyperlipidemia, insulin resistance, and hypertension within weeks to months of initiation [27].

These effects are particularly relevant in patients with preexisting coronary disease or metabolic syndrome and may accelerate atherosclerotic progression in long-term survivors.

Clinical implication: Early lipid and glucose monitoring should be integrated into cardio-oncology surveillance protocols for patients receiving metabolically active therapies.

### 4.4 Autonomic Dysfunction and Delayed Radiation Effects

Radiation therapy, proteasome inhibitors, and certain TKIs have been linked to autonomic dysfunction manifesting as impaired heart-rate recovery, sinus node dysfunction, and orthostatic intolerance [28].

Reduced heart-rate recovery (≤12 beats per minute at 1 minute post-exercise) reflects impaired parasympathetic reactivation and is associated with increased mortality in cancer survivors, particularly those exposed to mediastinal radiation.

### 4.5 Microvascular and Endothelial Preservation Paradigms

Endothelial dysfunction has emerged as a unifying mechanism across multiple modern therapies, including VEGF inhibitors, proteasome inhibitors, and platinum-based chemotherapy. Capillary rarefaction, nitric oxide depletion, and prothrombotic signaling contribute to heart failure with preserved ejection fraction, accelerated coronary disease, and arterial thrombosis [29].

This paradigm helps explain why patients may develop ischemic symptoms despite angiographically normal coronary arteries and underscores the limitations of traditional ischemia testing in this population.

## 5. Monitoring, Prevention, and Management Paradigms

Effective management of cancer therapy–related cardiovascular toxicity requires a proactive, risk-adapted strategy that integrates baseline cardiovascular assessment, longitudinal surveillance, and early intervention. Contemporary cardio-oncology practice has shifted from reactive treatment of overt heart failure toward prevention and early detection of subclinical myocardial injury, recognizing that timely intervention can mitigate irreversible damage and improve long-term outcomes [8,30].

### 5.1 Baseline Cardiovascular Risk Stratification

Baseline cardiovascular risk assessment is essential prior to initiation of potentially cardiotoxic cancer therapies. Key determinants include patient-related factors (age, hypertension, diabetes, dyslipidemia, prior cardiovascular disease), cancer-related factors (type, stage, planned multimodality therapy), and treatment-related factors (cumulative anthracycline dose, mediastinal radiation, combination regimens) [8].

Structured risk stratification tools endorsed by ESC–ICOS guidelines categorize patients into low, moderate, high, or very high cardiovascular risk groups, informing surveillance intensity and preventive strategies. High-risk patients derive the greatest benefit from early cardioprotective interventions and intensified monitoring.

### 5.2 Imaging Surveillance

Transthoracic echocardiography remains the cornerstone of cardiovascular surveillance in cardio-oncology. Serial assessment of left ventricular ejection fraction is complemented by global longitudinal strain (GLS), which enables detection of subclinical myocardial dysfunction before overt ejection fraction (EF) decline. A relative reduction in GLS exceeding 15% from baseline is considered abnormal and predictive of future cardiotoxicity [31].

Cardiac magnetic resonance imaging plays a critical role in selected scenarios, including suspected myocarditis, infiltrative cardiomyopathy, radiation-induced heart disease, and unexplained ventricular dysfunction. Tissue characterization with T1 mapping, T2 mapping, and late gadolinium enhancement allows differentiation between inflammation, fibrosis, edema, and necrosis, informing both diagnosis and prognosis [32].

### 5.3 Biomarker-Based Monitoring

Circulating biomarkers provide complementary insight into myocardial injury and hemodynamic stress. Cardiac troponins reflect cardiomyocyte injury and are particularly useful during anthracycline therapy and ICIs treatment. Persistent troponin elevation identifies patients at heightened risk for subsequent ventricular dysfunction [33].

Natriuretic peptides such as B-type natriuretic peptide (BNP) and N-terminal pro–B-type natriuretic peptide (NT-proBNP), reflect myocardial wall stress and volume overload. While less specific for early injury, rising levels during therapy may signal evolving heart failure or CRS-related myocardial stunning. Biomarker-guided strategies are most effective when integrated with imaging findings rather than used in isolation.

### 5.4 Pharmacologic Prevention and Cardioprotection

Neurohormonal blockade with angiotensin-converting enzyme inhibitors, angiotensin receptor blockers, beta-blockers, and emerging evidence supporting sodium-glucose cotransporter-2 (SGLT2) inhibitors forms the foundation of cardioprotective therapy in high-risk patients. Meta-analytic evidence suggests that SGLT2 inhibitors reduce the risk of chemotherapy-induced cardiac dysfunction and may represent a promising cardioprotective strategy in selected high-risk patients, although prospective randomized studies are still needed [34]. Fig. 2 outlines the ESC–ICOS-aligned framework for risk assessment, surveillance, and management of CTRCD.

Dexrazoxane remains the only U.S. Food and Drug Administration (FDA)-approved cardioprotective agent specifically indicated for anthracycline-induced cardiotoxicity and is recommended in patients receiving high cumulative doses or with preexisting cardiac dysfunction. Liposomal anthracycline formulations may further reduce myocardial exposure by lowering peak free-drug concentrations [35].

### 5.5 Therapy-Specific Management Algorithms

Management strategies must be tailored to the offending therapy and toxicity phenotype. In patients who develop cancer therapy–related left ventricular dysfunction, guideline-directed medical therapy (GDMT), including beta-blockers, renin–angiotensin system inhibitors (angiotensin-converting enzyme inhibitors, angiotensin receptor blockers, or angiotensin receptor–neprilysin inhibitors), mineralocorticoid receptor antagonists, and SGLT2 inhibitors, should be initiated and optimized as tolerated, in conjunction with therapy-specific oncologic management [8,34].

Anthracycline-induced cardiomyopathy is characterized by irreversible myocardial injury, and rechallenge after EF recovery carries substantial risk of rapid decompensation. In contrast, HER2-targeted therapy–related cardiomyopathy is typically reversible, permitting temporary treatment interruption and subsequent resumption once EF recovers [24].

ICIs–associated myocarditis is a potentially life-threatening cardiovascular toxicity. Current ESC–ICOS guidelines recommend prompt initiation of high-dose intravenous corticosteroids when ICI-associated myocarditis is strongly suspected, without delaying treatment while confirmatory evaluation is pursued [8]. Steroid-refractory cases may require escalation to additional immunosuppressive therapy, including mycophenolate mofetil or antithymocyte globulin [6,8].

CAR-T–associated cardiovascular dysfunction is primarily driven by cytokine release syndrome and responds to interleukin-6 (IL-6) blockade with tocilizumab, with corticosteroids reserved for refractory cases. Available evidence suggests that isolated troponin elevation should not, by itself, preclude tocilizumab therapy when cytokine release syndrome is clinically suspected [16].

### 5.6 Long-Term Survivorship and Cardiovascular Risk Modification

Cancer survivors remain at elevated cardiovascular risk long after completion of therapy. Survivorship care should emphasize aggressive management of traditional cardiovascular risk factors, including hypertension, dyslipidemia, diabetes, obesity, and physical inactivity. Radiation-exposed survivors require lifelong surveillance for coronary artery disease, valvular dysfunction, and conduction abnormalities [3].

Exercise training improves functional capacity, autonomic balance, and cardiometabolic health and should be incorporated into survivorship programs whenever feasible. Multidisciplinary collaboration between cardiology, oncology, and primary care is essential to ensure continuity of care across the cancer continuum.

## 6. Radiation-Induced Heart Disease

Radiation-induced heart disease (RIHD) refers to a broad spectrum of cardiovascular complications resulting from thoracic radiation therapy and is a leading cause of non-malignancy–related morbidity and mortality in cancer survivors, particularly those treated for Hodgkin lymphoma, breast, lung, and esophageal malignancies [36].

### 6.1 Clinical Manifestations and Latency

RIHD typically presents as a delayed “legacy effect”, with clinical manifestations emerging years to decades after exposure. Coronary artery disease is the most common manifestation, occurring in up to 85% of patients in high-risk cohorts, and is characterized by accelerated atherosclerosis with a predilection for proximal and ostial lesions; myocardial infarction may be clinically silent due to radiation-induced autonomic dysfunction. Valvular heart disease develops through progressive leaflet thickening and calcification, most commonly affecting the aortic and mitral valves, with regurgitation often preceding late stenosis after two or more decades. Myocardial involvement manifests as restrictive cardiomyopathy with diastolic dysfunction and heart failure with preserved ejection fraction (HFpEF) physiology due to diffuse fibrosis. Pericardial disease ranges from acute pericarditis to chronic constrictive pericarditis, the latter associated with poor surgical outcomes. Conduction abnormalities, including right bundle branch block, QT prolongation, and sinus node dysfunction, reflect progressive fibrosis of the conduction system [37].

### 6.2 Pathophysiology

The central initiating event in RIHD is endothelial injury induced by ionizing radiation. In the acute phase, radiation generates reactive oxygen species, resulting in DNA damage and activation of inflammatory signaling pathways, including NF-κB, interleukin-6, and tumor necrosis factor-α. In the chronic phase, persistent inflammation leads to myofibroblast activation and progressive collagen deposition, producing fibrosis of the myocardium, valves, pericardium, and vasculature. Concurrent microvascular rarefaction and endothelial dysfunction impair myocardial perfusion and accelerate macrovascular atherosclerosis, providing a unifying mechanism for both ischemic and nonischemic manifestations [37].

### 6.3 Radiation Dose and Interaction With Anthracyclines

The risk of RIHD is dose-dependent and correlates with mean heart dose (MHD), with no clearly defined safe threshold. Epidemiologic data demonstrate an approximate 7% relative increase in major coronary events per 1 Gy increase in MHD, with risk beginning within the first decade and persisting lifelong. Higher risk is observed with MHD >5 Gy, left-sided chest irradiation, larger irradiated cardiac volumes, and anterior mediastinal exposure [36].

Cardiotoxicity is further amplified by combined exposure to anthracyclines. Anthracycline-related risk is cumulative, increasing substantially at doxorubicin doses ≥250 mg/m^2^ (or equivalent), and when combined with radiation results in synergistic myocardial injury through complementary mechanisms of oxidative stress, endothelial dysfunction, and fibrosis. Patients receiving both therapies have a significantly higher incidence of cardiomyopathy, heart failure, and ischemic events compared with either exposure alone [28,35].

### 6.4 Management and Surveillance

Management of RIHD is challenging due to radiation-induced fibrosis and mediastinal scarring, which create a “hostile chest” for surgical intervention. Current recommendations support cardiovascular screening with transthoracic echocardiography beginning approximately 5 (high risk)–10 years (low risk) after radiation therapy (RT), or earlier in high-risk individuals, with periodic follow-up thereafter.

In patients with valvular disease, transcatheter approaches such as transcatheter aortic valve replacement are generally preferred over surgical valve replacement due to increased operative risk. Similarly, percutaneous coronary intervention is often favored over coronary artery bypass grafting, as prior radiation may compromise conduit vessels, particularly the internal mammary arteries. Preventive strategies, including dose minimization, deep inspiration breath-hold techniques, and proton therapy, are essential to reduce MHD and mitigate long-term cardiovascular risk [38].

## 7. Cardiac Amyloidosis in the Cardio-Oncology Spectrum

Cardiac amyloidosis (CA) is an infiltrative cardiomyopathy caused by extracellular deposition of misfolded protein fibrils within the myocardium, resulting in restrictive physiology, HFpEF, arrhythmias, and conduction disease. Within cardio-oncology, CA is relevant because light-chain (AL) amyloidosis is a true oncologic disorder, while transthyretin (ATTR) amyloidosis frequently coexists in older cancer populations and can overlap clinically [39].

### 7.1 Classification: Light-Chain (AL) vs. Transthyretin (ATTR) Amyloidosis

More than 30 amyloidogenic proteins have been identified; however, AL and ATTR account for the vast majority of clinically significant cardiac amyloidosis, with other forms being rare and infrequently involving the heart.

AL amyloidosis is derived from immunoglobulin κ or λ light chains due to a plasma cell dyscrasia. It is rapidly progressive with direct myocardial toxicity and high early mortality if untreated, requiring urgent hematologic therapy [40].

ATTR amyloidosis results from misfolded transthyretin (TTR) and includes wild-type (ATTRwt) and variant/hereditary (ATTRv) forms. Terminology including hereditary transthyretin amyloidosis (hATTR), ATTRv, and mutant transthyretin amyloidosis (ATTRm) (older term) all refer to mutation-associated disease. ATTR generally has slower progression and is now treatable with disease-modifying therapies [41].

### 7.2 Red-Flag Clinical and Multi-Modality Imaging Features

Clinical suspicion for CA should arise with increased LV wall thickness disproportionate to loading conditions, especially in HFpEF. ECG may show low voltage or pseudo-infarct patterns, and echocardiography often demonstrates apical sparing on GLS. Persistent biomarker elevation, atrial fibrillation (AF), conduction disease, and orthostatic symptoms are common. Extracardiac features such as CTS, neuropathy, proteinuria, macroglossia, or periorbital purpura provide additional clues.

Coexistent severe aortic stenosis (AS) is increasingly recognized, with ATTR-CA identified in approximately 10–16% of patients undergoing transcatheter aortic valve replacement (TAVR), particularly in older adults, which has important diagnostic and prognostic implications [42].

### 7.3 Structured Diagnostic Approach and Systematic Exclusion of AL

A structured diagnostic approach is essential to accurately identify cardiac amyloidosis and distinguish between AL and ATTR subtypes [42,43,44]. Clinical suspicion should be raised in patients presenting with unexplained heart failure, particularly heart failure with preserved ejection fraction, increased left ventricular wall thickness (>12 mm) disproportionate to loading conditions, carpal tunnel syndrome, or a low voltage-to-mass ratio on electrocardiography.

Echocardiography serves as the first-line imaging modality and typically demonstrates increased ventricular wall thickness, biatrial enlargement, and diastolic dysfunction. A reduction in global longitudinal strain with relative apical sparing represents a characteristic pattern that supports the diagnosis. Cardiac magnetic resonance imaging provides further tissue characterization, with late gadolinium enhancement often demonstrating diffuse subendocardial or transmural involvement, along with elevated native T1 values and increased extracellular volume, reflecting amyloid infiltration [43].

A critical early step in the diagnostic pathway is the mandatory exclusion of AL amyloidosis, which represents a rapidly progressive medical emergency. This requires a combination of serum free light chain analysis, assessing for abnormal kappa-to-lambda ratio, along with serum and urine immunofixation. Detection of a monoclonal protein should prompt urgent hematologic evaluation to confirm or exclude AL amyloidosis [40].

In the absence of monoclonal protein, bone scintigraphy using technetium-labeled tracers such as technetium-99m pyrophosphate (Tc-99m PYP) can establish the diagnosis of ATTR cardiac amyloidosis without the need for biopsy. A Perugini grade of 2 or 3, indicating myocardial uptake equal to or greater than rib uptake, is highly specific for ATTR when AL has been excluded [41].

Following confirmation of ATTR amyloidosis, genetic testing is required to distinguish hereditary from wild-type disease. Pathogenic variants in the transthyretin gene define hereditary ATTR, with the valine-to-isoleucine substitution at position 122 (Val122Ile) mutation being the most common variant associated with cardiac involvement, particularly among individuals of African or Afro-Caribbean ancestry. Identification of a pathogenic mutation has important implications for family screening and genetic counseling.

Tissue biopsy remains necessary in selected scenarios, including cases with positive monoclonal protein studies, equivocal imaging findings, or discordant clinical data. Biopsy of affected organs or surrogate sites such as abdominal fat pad, rectum, or endomyocardial, followed by Congo red staining and protein typing using mass spectrometry, provides definitive diagnosis and subtype confirmation [39].

### 7.4 Thromboembolic Risk and Atrial Mechanical Dysfunction

Cardiac amyloidosis is associated with a high risk of intracardiac thrombus and systemic embolism due to atrial mechanical dysfunction, restrictive physiology, and AF, and thrombus may occur even in sinus rhythm. In patients with cardiac amyloidosis and AF, anticoagulation is recommended regardless of the CHA_2_DS_2_-VASc score. The thromboembolic risk is particularly high in AL amyloidosis and is not adequately captured by conventional risk scores. Because intracardiac thrombus may occur despite therapeutic anticoagulation, transesophageal echocardiography (TEE) should be performed before cardioversion regardless of anticoagulation status [42].

### 7.5 Disease-Modifying Therapies

In ATTR amyloidosis, disease-modifying therapies include TTR stabilizers (tafamidis and acoramidis) and TTR gene-silencing agents (vutrisiran, patisiran, inotersen, and eplontersen), which act by stabilizing the TTR tetramer or reducing hepatic TTR production [42,43,45]. Currently, tafamidis, acoramidis, and vutrisiran are FDA-approved specifically for transthyretin amyloid cardiomyopathy (ATTR-CM) (Table 2, Ref. [45,46,47]) [42]. AL amyloidosis requires urgent clone-directed hematologic therapy targeting the plasma cell disorder, as cardiac involvement is a key determinant of prognosis [44].

**Table 2.** **Disease-modifying therapies for ATTR cardiomyopathy**.

Drug	Class	Mechanism	Dose	Key trial
Tafamidis	TTR stabilizer	Reduces tetramer dissociation and amyloid formation	61 mg PO once daily	ATTR-ACT [45]
Acoramidis	TTR stabilizer	High-affinity TTR tetramer stabilizer, reducing tetramer dissociation and amyloid formation	712 mg PO twice daily	ATTRibute-CM [46]
Vutrisiran	siRNA TTR silencer	Reduces hepatic TTR production through RNA interference–mediated degradation of TTR mRNA	25 mg SC every 3 months	HELIOS-B [47]

Abbreviations: ATTR-ACT, Transthyretin Amyloidosis Cardiomyopathy Clinical Trial; ATTRibute-CM, A Study to Evaluate the Efficacy and Safety of AG10 in Subjects With Amyloid Cardiomyopathy; mRNA, messenger ribonucleic acid; PO, per os (orally); RNA, ribonucleic acid; SC, subcutaneous; siRNA, small interfering ribonucleic acid; TTR, transthyretin.

### 7.6 Monitoring

In ATTR-CM, recently proposed criteria assess disease progression across clinical, biomarker, and functional domains, including HF-related hospitalization, outpatient diuretic intensification, changes in NT-proBNP and eGFR (estimated glomerular filtration rate), decline in 6-minute walk test (6MWT) performance, and worsening Kansas City Cardiomyopathy Questionnaire (KCCQ) score or New York Heart Association (NYHA) functional class [48].

## 8. Future Directions, Artificial Intelligence, and Knowledge Gaps

Despite substantial advances in cardio-oncology, important gaps remain in risk prediction, early detection, and long-term management of cancer therapy–related cardiovascular toxicity. Current risk stratification models rely largely on clinical factors and treatment exposure but lack precision at the individual patient level. Emerging data suggest that incorporation of genetic susceptibility, including variants affecting myocardial resilience such as titin truncating mutations, may improve risk prediction and enable personalized surveillance strategies [49].

Advances in imaging and biomarker technologies offer opportunities for earlier detection of subclinical myocardial injury; however, optimal thresholds for intervention remain incompletely defined. The integration of multimodality imaging with serial biomarker assessment requires further standardization, particularly in patients receiving novel therapies with heterogeneous toxicity profiles [50].

Artificial intelligence and machine learning approaches are increasingly being explored to enhance pattern recognition across imaging, electrocardiographic, and biomarker data. These tools have shown potential to identify early signals of cardiotoxicity and may eventually guide individualized monitoring strategies, although prospective validation and clinical integration remain ongoing challenges [51].

Long-term cardiovascular outcomes associated with newer oncologic agents, including ICIs, targeted therapies, and cellular therapies, are not yet fully characterized. Delayed cardiomyopathy, persistent inflammatory states, and vascular dysfunction represent emerging concerns that require longitudinal follow-up and dedicated survivorship frameworks [24].

Access to specialized cardio-oncology care remains variable across healthcare systems, limiting implementation of guideline-based surveillance and management. Expansion of multidisciplinary care models, standardized care pathways, and education of both cardiology and oncology providers will be essential to bridge this gap and improve patient outcomes [52].

## 9. High-Yield Clinical Pearls in Cardio-Oncology

Several cardiovascular syndromes encountered in cardio-oncology require rapid recognition and nuanced management that may not be fully captured by therapy-specific toxicity frameworks. Table 3 summarizes selected high-yield clinical patterns, common diagnostic or management pitfalls, and practical take-home points relevant to contemporary cardio-oncology practice.

**Table 3.** **Selected diagnostic and management pearls in cardio-oncology**.

Clinical pearl	Clinical clues / pattern	Diagnostic or Management pitfall	take-home pearl
Constrictive pericarditis post-RT	Edema, ascites, increased JVP, Kussmaul sign; remote thoracic RT (years–decades).	TTE may be inconclusive; cirrhosis and restrictive CMP can mimic the presentation. Use CMR / CT and invasive hemodynamics when noninvasive findings remain equivocal.	Respiratory LV / RV systolic pressure discordance reflects ventricular interdependence and supports constriction.
Hostile chest after RT	Mediastinal fibrosis, adhesions, porcelain aorta, compromised LIMA / RIMA conduits.	Surgical re-entry carries increased risk from fibrosis, poor wound healing, calcification, and limited bypass conduits.	Prior chest RT often favors a percutaneous strategy when technically feasible: TAVR in many intermediate / high-risk patients and PCI when CABG risk or conduit limitations are substantial. Heart Team assessment remains essential.
Cancer-associated thrombosis	GI, GU, GYN malignancies; IMiDs + dexamethasone; indwelling CVC; active/metastatic cancer.	Stopping AC too early despite persistent cancer- or CVC-related risk; assuming all ambulatory patients require prophylaxis.	Khorana ≥2 → consider thromboprophylaxis if bleeding risk is acceptable. CVC-DVT → AC for ≥3 mo and consider continuation while the CVC remains. VTE risk persists during active cancer / therapy.
Nonbacterial thrombotic endocarditis	Active malignancy, often adenocarcinoma; recurrent or multi-territory emboli involving brain, spleen, kidney, or limbs.	Negative blood cultures and nondiagnostic TTE may delay diagnosis. Small friable vegetations are better detected with TEE.	Sterile platelet-fibrin vegetations typically occur on left-sided valves without valve destruction. Consider NBTE in recurrent embolic events without evidence of infection.
Carcinoid Heart disease	Flushing, secretory diarrhea, hepatomegaly; severe TR ± pulmonic regurgitation / stenosis.	Left-sided disease may be missed. Evaluate for PFO, bronchial NET, pulmonary metastasis, or very high circulating mediator burden.	Pulmonary metabolism of vasoactive mediators usually protects the left heart, producing predominantly right-sided valvular disease.
Loeffler endocarditis / cardiac HES	Hypereosinophilia; apical thrombus / obliteration; systemic emboli; progression from necrotic → thrombotic → fibrotic disease.	Delayed recognition permits irreversible endomyocardial fibrosis. Therapy should be directed by the underlying HES subtype.	CMR defines thrombus and subendocardial fibrosis. Corticosteroids are commonly used for eosinophilic disease; imatinib is indicated for *FIP1L1-PDGFRA*–positive disease; AC when intracardiac thrombus or another indication is present.
CIED management during RT	PM / ICD with thoracic, breast, mediastinal, or nodal RT; pacing dependence; neutron-producing radiation.	Routine generator relocation may expose patients to unnecessary procedural risk. Device dose alone does not determine risk.	Minimize CIED exposure, ideally <2 Gy and ≤5 Gy when feasible; avoid neutron-producing treatment when possible. High-energy photons >10 MV, proton therapy, pacing dependence, and ICDs increase risk.
Malignant pericardial tamponade	Large neoplastic effusion; dyspnea, increased JVP, pulsus paradoxus, RA / RV diastolic collapse; commonly lung/breast malignancy, lymphoma, melanoma.	Isolated needle drainage has a high recurrence rate. Malignant effusions recur in >50% of cases.	Tamponade → urgent echo-guided pericardiocentesis. Leave a drain until output is <30 mL/day; consider extended drainage or pericardial window for recurrent neoplastic effusion.
Thrombocytopenia: ACS / PCI and VTE	STEMI / NSTE-ACS or acute VTE with cancer- or therapy-related thrombocytopenia.	Thrombocytopenia does not automatically preclude invasive treatment or AC. Bleeding and thrombotic risks must both be considered.	Radial access is preferred when feasible. Expert guidance supports PCI at platelets ≥30K/µL and CABG at ≥50K/µL; use reduced UFH when platelets are <50K. VTE AC intensity should be individualized by platelet count, clot severity, and bleeding risk.
Fluoropyrimidine vasospasm & overdose	Rest angina, ischemic ECG changes, ACS, or ventricular arrhythmias during 5-FU / capecitabine.	Automatic rechallenge without CV assessment may cause recurrent ischemia. Uridine triacetate is not routine treatment for uncomplicated vasospasm.	If rechallenge is necessary, exclude severe CAD and consider monitored rechallenge with nitrate / CCB prophylaxis after MDT discussion. Uridine triacetate should be given within 96 h for overdose or early severe / life-threatening toxicity.
Cardiopulmonary overlap syndromes	Dasatinib → pleural effusion / PAH; gemcitabine → capillary leak/pulmonary edema; cancer itself may cause PE or pulmonary vascular disease.	Dyspnea may be incorrectly attributed to LV HF alone. Empiric diuresis may not address the underlying pulmonary vascular or endothelial process.	Evaluate cardiac and pulmonary causes together. TTE is first-line; RHC is appropriate when PH mechanism remains uncertain and distinguishing pre- from post-capillary PH will alter management.

Abbreviations: AC, anticoagulation; ACS, acute coronary syndrome; CABG, coronary artery bypass grafting; CCB, calcium channel blocker; CIED, cardiac implantable electronic device; CMR, cardiac magnetic resonance; CMP, cardiomyopathy; CT, computed tomography; CV, cardiovascular; CVC, central venous catheter; DVT, deep vein thrombosis; GI, gastrointestinal; GU, genitourinary; GYN, gynecologic; HES, hypereosinophilic syndrome; ICD, implantable cardioverter-defibrillator; IMiDs, immunomodulatory drugs; JVP, jugular venous pressure; LIMA, left internal mammary artery; MDT, multidisciplinary team; NET, neuroendocrine tumor; NBTE, nonbacterial thrombotic endocarditis; NSTE-ACS, non-ST-segment elevation acute coronary syndrome; PAH, pulmonary arterial hypertension; PCI, percutaneous coronary intervention; PE, pulmonary embolism; PFO, patent foramen ovale; PH, pulmonary hypertension; PM, pacemaker; RA, right atrium; RHC, right heart catheterization; RIMA, right internal mammary artery; RT, radiation therapy; RV, right ventricle; SAVR, surgical aortic valve replacement; STEMI, ST-segment elevation myocardial infarction; TAVR, transcatheter aortic valve replacement; TEE, transesophageal echocardiography; TR, tricuspid regurgitation; TTE, transthoracic echocardiography; UFH, unfractionated heparin; VTE, venous thromboembolism; FIP1L1-PDGFRA, FIP1-like-1–platelet-derived growth factor receptor alpha (fusion gene/tyrosine kinase); MV, megavolt.

## 10. Conclusions

Cancer therapy-related cardiovascular toxicity represents a dynamic, heterogeneous spectrum extending far beyond left ventricular ejection fraction decline. Its clinical course reflects the interplay of drug-specific mechanisms, cumulative and multimodality exposures, patient-level baseline risk, and time from therapy. Modern cardio-oncology care must be anticipatory, mechanism-informed, and risk-adapted. This requires pairing baseline risk stratification with integrated biomarker and imaging surveillance to drive phenotype-specific interventions. Expanding targeted agents, cellular and immune therapies, and thoracic radiotherapy demand continuous vigilance for inflammatory, vascular, electrophysiologic, and metabolic sequelae across the cancer continuum. Advancing this field requires validating therapy-specific monitoring thresholds, broadening access to specialized multidisciplinary teams, and responsibly integrating molecular and artificial intelligence tools. Ultimately, the goal is cardiovascular health preservation to maximize oncologic efficacy and overall survival.
